# Survival of Older Adults Choosing Dialysis or Conservative Kidney Management, Stratified by Suitability for Dialysis

**DOI:** 10.1016/j.xkme.2026.101430

**Published:** 2026-06-12

**Authors:** Micha Jongejan, Marjolijn van Buren, Wouter R. Verberne, Gurbey Ocak, Willem Jan W. Bos

**Affiliations:** 1 Department of Internal Medicine, Leiden University Medical Center, Leiden, the Netherlands; 2 Department of Internal Medicine, Haga Hospital, The Hague, the Netherlands; 3 Department of Internal Medicine, St. Antonius Hospital, Nieuwegein, the Netherlands

**Keywords:** Conservative care, conservative kidney management, dialysis, elderly, kidney failure, mortality, renal failure, shared decision making, survival

## Abstract

**Rationale & Objective:**

Previous studies indicate survival benefit for dialysis over conservative kidney management (CKM), which attenuates in patients ≥80 years or with high comorbidity. Most studies did not differentiate dialysis suitability among those choosing CKM, potentially biasing outcomes. We aimed to describe survival outcomes in patients choosing dialysis, patients suitable for dialysis who chose CKM, and patients less suitable for dialysis who chose CKM.

**Study design:**

Single-center cohort study.

**Setting & Participants:**

We included patients aged ≥65 years with a documented treatment decision for dialysis or CKM between 2017 and 2024.

**Exposure:**

Chosen treatment (dialysis or CKM). Patients choosing CKM were classified as suitable or less suitable for dialysis, based on the treating nephrologist’s.

**Outcome:**

The primary outcome was all-cause mortality.

**Analytical Approach:**

Descriptive, using Kaplan–Meier estimates and Cox proportional hazard models without adjustment for confounding.

**Results:**

Median survival from treatment decision was 53 (dialysis), 32 (CKM, suitable for dialysis), and 18 months (CKM, less suitable for dialysis), with unadjusted hazard ratios of 2.11 (CKM, suitable for dialysis vs dialysis; 95% CI 1.59-2.80) and 3.23 (less suitable for dialysis vs dialysis; 95% CI 2.47-4.23). Among patients aged ≥80 years, hazard ratios were 1.89 (0.99-1.74) for CKM suitable for dialysis versus dialysis and 2.35 (1.31-4.64) for CKM less suitable for dialysis versus dialysis. Thirteen percent of patients who initially chose dialysis switched to CKM, whereas 1% transitioned from CKM to dialysis.

**Limitations:**

Subjectivity in dialysis suitability assessment.

**Conclusions:**

In this cohort of older patients with kidney failure, survival outcomes in patients choosing CKM suitable for dialysis were longer than previously reported. These findings highlight the importance of distinguishing dialysis suitability when evaluating survival outcomes in CKM and dialysis. This supports more individualized shared decision making in older adults with advanced kidney disease.

## Plain-Language Summary

Older adults with advanced kidney disease often face a difficult decision whether to choose dialysis or conservative kidney management (CKM). Earlier studies did not clarify whether patients opting for CKM were medically eligible for dialysis, making results less applicable for people who face a genuine choice between treatments. This study described survival outcomes in people aged 65 years and older. People who chose dialysis lived about 53 months after making their treatment decision, people who chose CKM and were considered suitable for dialysis lived about 32 months, and people who chose CKM and were considered less suitable for dialysis lived about 18 months. These findings support individualized shared decision making and highlight the importance of considering dialysis suitability.

Older adults approaching kidney failure face a challenging decision when considering treatment options. These options include kidney transplantation, hemodialysis (HD), peritoneal dialysis (PD), or conservative kidney management (CKM). Because older or frail patients are often ineligible for transplantation, they must weigh the benefits and burdens of dialysis and CKM. CKM is a comprehensive approach that focuses on medical management, symptom control, and multidisciplinary support to improve quality of life.^1^^,^^2^ Qualitative studies described that patients perceive dialysis as the default treatment and often associate choosing CKM with imminent death.^3^^,^^4^ Uncertainty regarding survival with CKM is also mentioned by nephrologists as a reason for not discussing CKM with patients.^5^ In this context, observational studies describing actual survival in patients choosing CKM provide valuable insights given that knowledge of survival and other outcomes is essential for shared decision making.^6^

Previous observational studies indicate a survival benefit for patients treated with dialysis, compared with those choosing CKM, although this benefit attenuates for patients over the age of 80 years or with higher comorbid conditions.^7^ Comparing survival outcomes between dialysis and CKM is particularly valuable for patients suitable for both treatment options. Prior studies often did not specify whether cohorts included patients considered suitable for dialysis.^8^, ^9^, ^10^, ^11^, ^12^, ^13^, ^14^ Including patients unsuitable for dialysis, who typically have higher comorbid conditions and a poorer functional status, will likely lower survival estimates.^15^ One study reported survival outcomes of patients choosing CKM, distinguishing between those who personally chose CKM and those for whom dialysis was deemed inappropriate by the nephrologist. For these 2 groups, median survival was 25 (interquartile range [IQR] 11-45) months and 6 (IQR 4-19) months, respectively, starting follow-up from a median eGFR of 14 mL/min/1.73 m^2^.^16^

After the initial treatment decision, patients may change their mind. In previous studies, the range of patients changing their decision from dialysis to CKM before treatment initiation is between 0% and 12.1%, whereas between 0% and 3.9% switch from CKM to dialysis.^17^ Data about treatment modality switches are only available within study settings with selected patients included in these studies, and there is a lack of real-world data on this topic.

We aimed to describe survival outcomes of older patients choosing dialysis, patients considered suitable for dialysis who chose CKM, and patients less suitable for dialysis. The secondary objective of this study is to describe the proportion of patients changing their treatment decision from dialysis to CKM or vice versa prior to dialysis initiation.

## Materials and Methods

### Study Design and Participants

We conducted a single-center cohort study using deidentified electronic health records (EHRs) obtained from St. Antonius Hospital, a nonacademic teaching hospital in the Netherlands with 5 nephrologists. The team is dedicated to shared decision making and has implemented a structured pathway for chronic kidney disease (CKD) and CKM. The EHR was searched for patients aged ≥65 years who received outpatient nephrology care in an advanced kidney care pathway and had a documented treatment decision for dialysis or CKM between January 1, 2017, and February 20, 2024. As part of this care pathway, the treating nephrologist assesses dialysis suitability for patients choosing CKM. This assessment is subjective and personalized, typically guided by the nephrologist’s clinical judgment. Factors such as comorbid conditions, daily functioning, and cognitive status are considered, though no standardized criteria currently exist. Based on this assessment, the nephrologist formulates the most suitable treatment, which is taken into account during counseling about the available treatment options and informs the subsequent shared decision making process. In this study, we refer to this distinction as “suitable for dialysis” versus “less suitable for dialysis”. Unless there were absolute contraindications for dialysis, patients who were considered less suitable were still involved in a shared decision-making process and given the opportunity to choose. Patients were categorized based on their initial treatment decision (dialysis or CKM) and stratified on their suitability for dialysis, in an intention-to-treat approach, irrespective of whether they ultimately commenced dialysis, changed their treatment decision, or died prior to dialysis initiation.

Patients without an explicit, documented treatment decision for dialysis or CKM were excluded, either because of acute kidney injury or because dialysis preparation and/or initiation occurred at a different health care facility. Patients were also excluded if their initial treatment decision was made before 2017, estimated glomerular filtration rate (eGFR) was ≥20 mL/min/1.73 m^2^ at time of treatment decision, treatment decision was made in a palliative care setting or if patients opted for pre-emptive transplantation or dialysis as a bridging therapy to transplantation.

Follow-up started at the time of initial treatment decision registration and continued until death, kidney transplantation, or end of follow-up on January 23 2025, whichever occurred first. Patients were censored if they underwent kidney transplantation.

### Counseling About Kidney Failure Treatment

The decision-making process takes place in a kidney care and education pathway. From an eGFR of 20 mL/min/1.73 m^2^ or lower, education about kidney replacement therapy (KRT) options and CKM is initiated by a multidisciplinary team. Since December 2020, this process is supplemented with a patient decision aid to enhance shared decision making.^18^^,^^19^ For patients choosing HD or PD, dialysis initiation typically occurs when eGFR reached 7 to 10 mL/min/1.73 m^2^. CKM comprises ongoing care by a multidisciplinary team of nephrologists, nurse practitioners, dietitians and medical social workers, focusing on symptom management and quality of life without dialysis. This included medical management of kidney disease complications, psychosocial support, and advance care planning.

### Data Collection

We collected patient demographics, comorbid conditions, laboratory results, the initial treatment modality decision, any subsequent changes in the chosen treatment modality, and the dates at which eGFR was below 20, 15, and 10 mL/min/1.73 m^2^. We excluded inpatient eGFR measurements taken during hospital admissions because such measurements are often influenced by temporary changes in kidney function, which may not accurately reflect the patient’s long-term kidney function. Outpatient eGFR measurements are considered more representative of the chronic kidney function over time. The eGFR was calculated using the Chronic Kidney Disease Epidemiology Collaboration (CKD-EPI) 2009.^20^ The dates of treatment decision registration and any subsequent changes were manually verified in the EHR. When documented, the reason for the change and whether it was initiated by the patient or the nephrologist were also recorded. Treatment modality changes after dialysis initiation including dialysis withdrawal were not collected. Dialysis suitability was documented for patients choosing CKM. Treating nephrologists retrospectively classified patients who chose dialysis as being considered suitable or less suitable for dialysis at the time the treatment decision was made, based on chart review and their clinical impressions. Considerations underlying nephrologists’ assessment of patients as less suitable for dialysis were identified through review of the EHR.

### Outcomes

The primary outcomes were the observed median, 1-year, 3-year, and 5-year survival of patients choosing dialysis, patients considered suitable for dialysis who chose CKM, as well as patients considered less suitable for dialysis, starting from the time of treatment decision. As a secondary outcome, we also described the proportion of patients who switched their treatment decision from dialysis to CKM and vice versa.

### Statistical Analysis

Variables are presented as mean (standard deviation), median (interquartile range), or numbers (percentage) when appropriate. Survival analyses were performed using Kaplan–Meier estimates and Cox proportional hazard model without adjustment for confounding. Given that the timing of treatment decisions may vary between groups, we conducted additional analyses with starting points starting at eGFR 15 and 10 mL/min/1.73 m^2^. Additionally, we conducted stratified analyses based on age (<80 versus ≥80 years). To provide an indicator of comparability in mortality risk between the groups before divergence in treatment pathways, we conducted a separate analysis in which follow-up was truncated at the first eGFR ≤ 10 mL/min/1.73 m^2^, ie, patients were censored when reaching eGFR ≤ 10 mL/min/1.73 m^2^. The eGFR threshold therefore represents a censoring criterion rather than an outcome and the outcome of interest in the model remained survival. Exploratory analyses were conducted to describe considerations documented in the EHR that may have contributed to nephrologists’ assessment of patients as less suitable for dialysis. These analyses were descriptive in nature. A sensitivity analysis was conducted to assess whether survival outcomes differed between patients choosing HD and PD, whereas in the main analysis, both groups were combined. Statistical analysis was performed using R version 4.5.1 (R Foundation for Statistical Computing, Vienna, Austria). The study was deemed not subject to the Medical Research Involving Human Subjects Act by Medical Research Ethics Committees United (#W24.042), and the requirement for informed consent was waived. This article adheres to the Strengthening the Reporting of Observational studies in Epidemiology (STROBE) guidelines.^21^

## Results

### Baseline Characteristics

**Figure 1:**
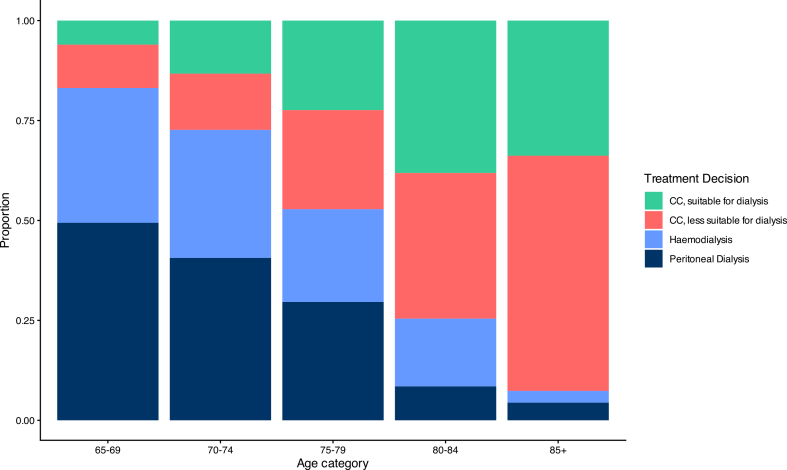
Distribution of treatment decisions by age category.

A total of 522 patients were included, with 263 patients choosing dialysis and 259 CKM, of whom 141 were considered less suitable for dialysis treatment by their treating nephrologist. Combinations of advanced age, frailty, and/or cardiac disease were the most frequently identified considerations for reduced dialysis suitability (Table S1). Ten patients who chose dialysis were retrospectively classified as less suitable. Of the patients choosing dialysis, 143 made an initial decision for PD and 120 for HD. Patients choosing dialysis were younger (mean ± standard deviation [SD]: 73 ± 5 years), whereas among patients choosing CKM, age did not differ between patients considered suitable or less suitable for dialysis (80 ± 6 years). As shown in Figure 1, the number of patients choosing CKM increased with age. At time of decision making, mean eGFR was 14.5 mL/min/1.73 m^2^ for patients choosing dialysis versus 16.6 mL/min/1.73 m^2^ for those choosing CKM, irrespective of suitability for dialysis (see Table 1). A flowchart with patients selection is shown in Figure S1.

**Table 1:** Baseline Characteristics of the Study Population

Characteristics	Total Study Population (n = 522)	Choosing Dialysis (n = 263)	Choosing Conservative Kidney Management	
			Considered Suitable for Dialysis (n = 118)	Considered less Suitable for Dialysis (n = 141)
Female sex	197 (37.7%)	102 (38.8%)	45 (38.1%)	50 (35.5%)
Age^a^ (y)	77 ± 6	73 ± 5	80 ± 5	80 ± 6
eGFR^a^	15.5 ± 5.1	14.5 ± 4.7	16.6 ± 4.8	16.6 ± 5.8
Proteinuria (UPCR, mg/mmol)^a^	0.2 ± 0.3	0.3 ± 0.3	0.2 ± 0.3	0.2 ± 0.3
Hemoglobin (g/dL)^a^	11.6 ± 1.6	11.6 ± 1.6	11.4 ± 1.6	11.6 ± 1.6
Serum albumin (g/dL)^a^	4.0 ± 0.4	4.0 ± 0.4	4.1 ± 0.4	4.0 ± 0.4

Abbreviations: eGFR, estimated glomerular filtration rate; HD, hemodialysis; PD, peritoneal dialysis; UPCR, urine total protein to creatinine ratio.

a At time of treatment decision.

### Survival

The median follow-up of this cohort was 24 months (IQR 13-43) from treatment decision. Among patients choosing dialysis, 120 (46%) patients died during follow-up, of whom 53 (20%) died before dialysis initiation. A total of 135 patients (51%) initiated dialysis during follow-up, and 21 patients (8%) underwent kidney transplantation. Among patients choosing CKM, 186 (72%) died during follow-up, with 112 of these deaths occurring before reaching eGFR ≤10 mL/min/1.73 m^2^ (43%).

**Figure 2:**
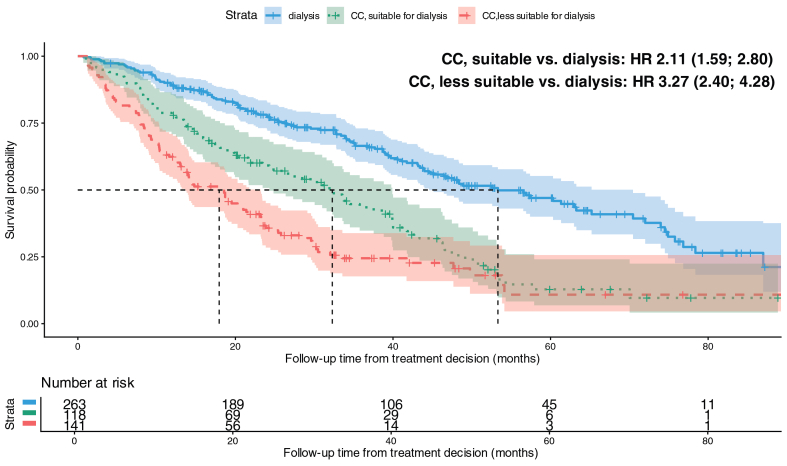
Kaplan–Meier estimates of survival from moment of treatment decision for patients choosing dialysis, patients suitable for dialysis who chose CKM and patients considered less suitable for dialysis.

As shown in Table 2 and Figure 2, patients choosing dialysis had a 1-year survival rate of 89.3% and a median survival of 53 months from the moment of treatment decision. Among patients choosing CKM, those considered suitable for dialysis had a 1-year survival rate of 78.0% and a median survival of 32 months, and patients less suitable for dialysis who chose CKM had a 1-year survival of 62.4% and a median survival of 18 months. The hazard ratio for mortality from the treatment decision was 2.11 (95% CI 1.59-2.80) for patients suitable for dialysis who chose CKM and 3.27 (95% CI 2.40-4.28) for those less suitable for dialysis compared with patients choosing dialysis. Similar patterns were observed when follow-up started from the moment eGFR reached 15 or 10 mL/min/1.73 m^2^ (Tables 2 and 3, Fig S2). Survival did not differ between patients choosing HD or PD (HR 1.03, 95% CI 0.68-1.54; Fig S3).

**Table 2:** Survival Rates from the Moment of Treatment Decision (Stratified by Age) as well as eGFR ≤15 and eGFR ≤10 mL/min/1.73m^2^

Start of Follow-up	1-Year Survival	3-Year Survival	5-Year Survival	Median Survival in Months (IQR)
Dialysis				
Moment of treatment decision	89.3%	66.5%	47.0%	53 (26-87)
65-79 y (n = 228)	89.4%	68.3%	47.7%	57 (31-87)
≥80 y (n = 35)	88.6%	54.9%	42.2%	43 (18-77)
eGFR ≤ 15 mL/min/1.73 m^2^ (n = 245)	93.8%	69.4%	47.5%	58 (33-99)
eGFR ≤ 10 mL/min/1.73 m^2^ (n = 188)	84.2%	57.6%	36.8%	41 (18-80)
CKM, suitable for dialysis				
Moment of treatment decision	78.0%	43.5%	12.9%	32 (13-48)
65-79 y (n = 50)	76.0%	43.5%	15.1%	32 (14-43)
≥80 y (n = 68)	79.4%	43.0%	11.6%	32 (13-51)
eGFR ≤ 15 mL/min/1.73 m^2^ (n = 85)	79.2%	44.5%	24.8%	31 (16-58)
eGFR ≤ 10 mL/min/1.73 m^2^ (n = 45)	50.4%	26.6%	NA	14 (8-42)
CKM, less suitable for dialysis				
Moment of treatment decision	62.4%	24.5%	10.9%	18 (8-34)
65-79 y (n = 58)	67.2%	28.2%	NA	20 (8-50)
≥80 y (n = 83)	59.0%	22.1%	12.6%	15 (8-32)
eGFR ≤ 15 mL/min/1.73m^2^ (n = 92)	73.4%	28.4%	21.6%	22 (11-50)
eGFR ≤ 10 mL/min/1.73m^2^ (n = 42)	41.5%	11.1%	NA	8 (6-19)

Abbreviations: CKM, conservative kidney management; eGFR, estimated glomerular filtration rate; NA, not applicable.

**Table 3:** Hazard Ratios of Mortality

Start of Follow-up	Hazard Ratio (95% CI)	Level of significance
CKM, suitable for dialysis versus dialysis		
Moment of treatment decision	2.11 (1.59-2.80)	*P* < 0.001
eGFR ≤15 mL/min/1.73 m^2^	2.15 (1.57-2.94)	*P* < 0.001
eGFR ≤10 mL/min/1.73 m^2^	2.59 (1.75-3.83)	*P* < 0.001
Treatment decision until eGFR 10 mL/min/1.73 m^2^	1.21 (0.92-1.60)	*P* = 0.18
CKM, less suitable for dialysis versus dialysis		
Moment of treatment decision	3.27 (2.40-4.28)	*P* < 0.001
eGFR ≤15 mL/min/1.73 m^2^	2.71 (2.00-3.68)	*P* < 0.001
eGFR ≤10 mL/min/1.73 m^2^	3.73 (2.52-5.52)	*P* < 0.001
Treatment decision until eGFR 10 mL/min/1.73 m^2^	1.54 (1.18-2.01)	*P* = 0.001

Abbreviations: CKM, conservative kidney management; CI, confidence interval; eGFR, estimated glomerular filtration rate.

**Figure 3:**
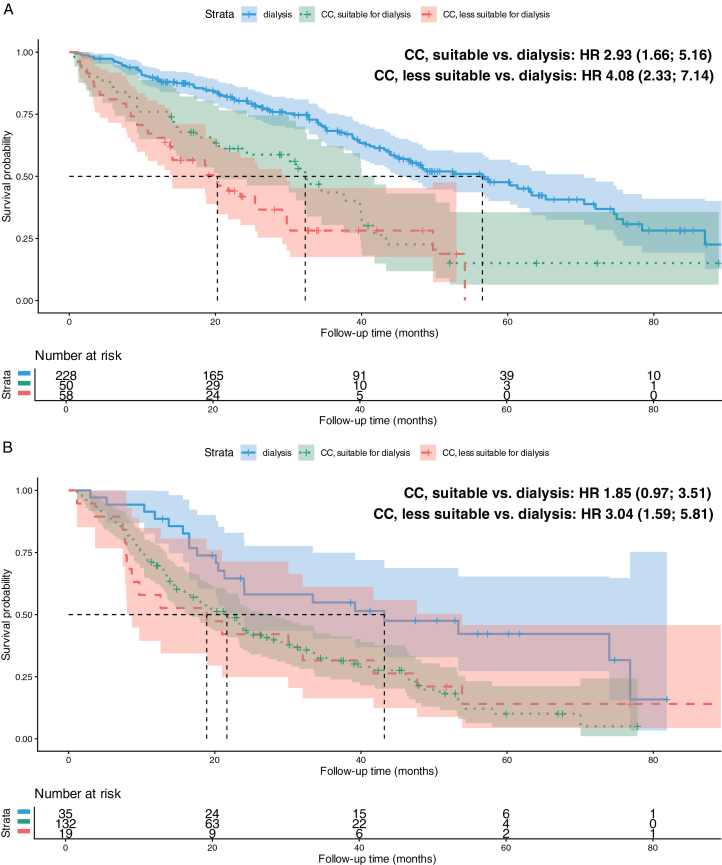
Kaplan–Meier estimates of survival from moment of treatment decision for patients choosing dialysis, patients considered suitable for dialysis who chose CKM and patients less suitable for dialysis stratified by age. (A) Age < 80 years. (B) Age ≥ 80 years.

**Figure 4:**
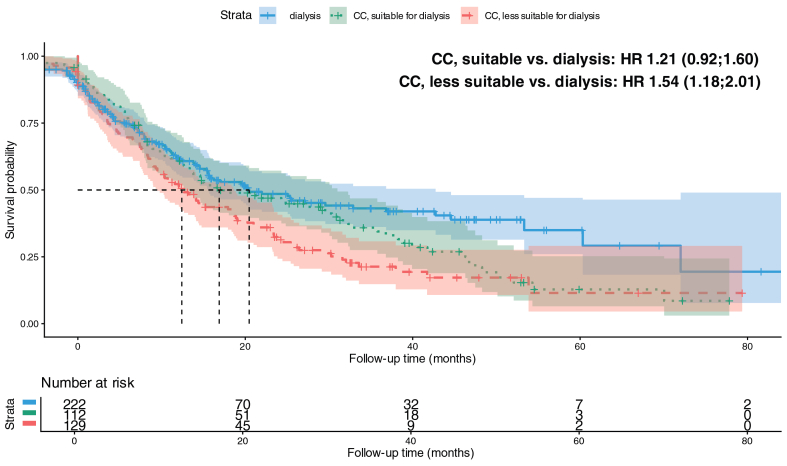
Kaplan–Meier curve of survival between time of treatment decision and 10 mL/min/1.73 m^2^ for patients choosing dialysis, patients considered suitable for dialysis who chose CKM and patients less suitable for dialysis.

Among patients aged ≥80 years, median survival from treatment decision was 43 months for patients choosing dialysis, 32 months for those considered suitable for dialysis who chose CKM, and 15 months for patients considered less suitable for dialysis who chose CKM (Table 2). Patients choosing CKM aged ≥80 years suitable for dialysis and those less suitable for dialysis had a mortality hazard ratio of 1.85 (95% CI 0.97-3.51) and 3.04 (95% CI 1.59-5.81) respectively, in comparison to patients choosing dialysis (Fig 3). From treatment decision until first eGFR ≤10 mL/min/1.73 m^2^, patients suitable for dialysis who chose CKM had a hazard ratio of 1.21 (0.92-1.60) for mortality compared with those choosing dialysis, whereas patients less suitable for dialysis who chose CKM had a hazard ratio of 1.54 (1.18-2.01) during this stage (Fig 4).

### Revision of Treatment Decision

A total of 37 patients changed their treatment decision after their initial decision; 34 (13%) patients changed their decision from dialysis to CKM and 3 (1%) vice versa. Among those who switched from dialysis to CKM, the median time between the initial and revised treatment decision was 665 days. In 18 of the 34 cases, the change was initiated by the patient. Common reasons for changing the treatment decision were worsening functional status and the emergence of additional comorbid conditions, including valvular heart disease, stroke, or dementia.

## Discussion

In this single-center cohort of older patients, patients choosing dialysis had the longest survival, followed by patients suitable for dialysis who chose CKM, and those less suitable for dialyses who chose CKM. These findings highlight the relevance of dialysis suitability and underscore the heterogeneity within the population choosing CKM. Furthermore, switching from dialysis to CKM occurred more frequently than switching from CKM to dialysis.

A systematic review by Voorend et al^7^ reported median survival from treatment decision ranging from 20 to 67 months for dialysis and from 6 to 31 months for CKM. In our study, survival of patients choosing dialysis fell within this range, whereas patients suitable for dialysis who chose CKM had a median survival of 32 months from moment of decision, which exceeds the range reported by Voorend et al.^7^ This may be explained by our distinction between dialysis suitability because patients less suitable for dialysis had a substantially shorter median survival of 18 months.

One prior study by Moranne et al^16^ categorized patients using dialysis suitability. However, despite using a similar stratification, we observed longer median survival across all groups (choosing dialysis: 40 vs 53 months, suitable for dialysis who chose CKM: 25 vs 32 months, less suitable for dialysis: 6 vs 18 months). These differences may reflect variations in dialysis suitability evaluation given that 12% of patients were considered less suitable for dialysis treatment in Moranne et al^16^ versus 25% in our study. Moreover, differences in study design may have contributed to the observed differences, including age criteria (≥75 years in Moranne et al^16^ vs ≥65 years in the current study) and timing of follow-up initiation (after at least 1 nephrology clinic visit with a median eGFR of 14 mL/min/1.73 m^2^ vs time of initial treatment decision).

Several other factors may explain the longer survival observed in our study. Growing awareness of CKM and enhanced understanding of its outcomes may have prompted more patients to choose CKM, potentially including more fit patients who consciously forego dialysis. In the current study, 50% of patients chose CKM. Although national or multicenter cohorts are lacking, this proportion appears relatively high.^22^ Recent studies reported rates of 7% to 40% of patients choosing CKM, suggesting differences in acceptance and implementation, beyond variation in study settings and study population characteristics.^8^, ^9^, ^10^, ^11^ The hospital where this study was conducted has actively implemented shared decision making and a structured care pathway for CKM, which likely contributes to a higher adoption.

Additionally, improvements in the quality of CKM over time may have contributed to longer survival in our study. This can be illustrated by comparing the survival outcomes of our study with those reported by Verberne et al,^14^ who included only patients considered suitable for dialysis in a study conducted at the same hospital. In this earlier study, the median survival from the treatment decision for CKM was 18 months, compared with 32 months in the current study.

In patients aged ≥80 years who chose dialysis, median survival was shorter than in those <80 years (43 vs 57 months). However, survival was longer than previously reported for this age group, with earlier studies finding median survival of 33 months from an eGFR ≤15 mL/min/1.73 m^2^ and 29 months from an eGFR ≤10 mL/min/1.73 m^2^.^23^^,^^24^ This may in part be because of ongoing improvements in survival.^25^ A similar age-related pattern was observed among patients considered less suitable for dialysis, whereas median survival did not differ by age group among patients suitable for dialysis who chose CKM.

In our study, 13% of patients who initially chose dialysis later revised their treatment decision to CKM, whereas 1% switched from CKM to dialysis. The decision to shift from dialysis to CKM was initiated either by the patient or the nephrologist, based on evolving personal preferences or in response to changes in health. These findings are in line with other studies that also report that more patients change their decision from dialysis to CKM than vice versa.^17^ As KRT counseling typically begins at an eGFR of 20 mL/min/1.73 m^2^, the time between treatment decision and dialysis initiation can span several years. During this period, patients grow older, and their health status, functional abilities, and priorities may shift. Previous research has shown that patients typically choose dialysis for life prolongation, whereas those choosing CKM prioritize quality of life.^26^ Our study showed that, with increasing age, patients more often chose CKM, reflecting a shift in priorities. This highlights the importance of ongoing discussions about treatment preferences because regular conversations can help patients to reconsider whether their chosen therapy still aligns with their goals of care.

A potential pitfall is to compare survival outcomes between the groups within this descriptive study. These groups are inherently different, making direct comparisons problematic and preventing causal attribution of any observed differences to the treatment. In this study, we have implemented several methodological approaches to explore differences between groups and to underscore the complexities inherent in observational studies, aiming to provide insights rather than direct comparisons.

First, we defined the start of follow-up at the moment of treatment decision rather than a specific eGFR threshold. If follow-up were to start at an eGFR before decision making (eg, eGFR 20), future information obtained during follow-up would be required to assign patients to treatment groups. This could introduce immortal time bias, as patients must survive long enough to be assigned to a group, potentially overestimating survival.^27^ Conversely, starting follow-up at an eGFR generally reached after the treatment decision (eg, eGFR 15 or 10) could introduce biases from depletion of susceptibles by excluding those who die before reaching that eGFR threshold. A potential limitation of using treatment decision as the start of follow-up is the variability between groups in eGFR at that moment, which may introduce lead-time bias, creating the impression of prolonged survival because of earlier cohort entry. In our study, patients choosing CKM had higher eGFR at the time of treatment decision than patients choosing dialysis. This finding contrasts with a previous qualitative study that described how health care professionals struggle to interpret a patient’s decision to forego dialysis and frequently revisit the decision, resulting in delays in decision registration.^10^ However, this difference becomes relevant only when comparing the groups; in our descriptive study, it reflects clinical practice at this hospital. In an ideal scenario, study entry would occur at a standardized disease severity level (eg, uniform eGFR threshold in CKD studies), with a defined time window for decision making, ensuring that suitability assessment, treatment assignment, and the start of follow-up coincide, in line with a randomized trial or target trial emulation study.^28^^,^^29^

Second, we stratified patients by dialysis suitability. When aiming to compare groups within a study, this approach helps to mitigate confounding by indication and account for unmeasured confounders such as frailty, malnutrition, cognitive impairment, and performance status. However, as dialysis suitability exists along a spectrum, and patients choosing dialysis may be fitter than those classified as suitable for dialysis who chose CKM, residual confounding persists. We chose not to adjust for measured confounders, for example, differences in age, as this would falsely give the impression of comparability between groups. Nevertheless, a previous study reported relatively small differences in geriatric impairments between older patients choosing dialysis and those choosing CKM, with 77% and 88% of patients in these groups, respectively, having at 2 or more geriatric impairments in a comprehensive geriatric assessment.^30^

Lastly, we examined survival between the treatment decision and the first eGFR ≤10 mL/min/1.73 m^2^, representing the period before actual treatment diverges. We observed similar survival in patients choosing dialysis and patients suitable for dialysis who chose CKM, which may suggest comparable mortality risk across groups at baseline. However, in this descriptive study, it remains uncertain whether this reflects baseline similarity, differences in disease severity at the time of treatment decision, or subtle differences in care received. In study designs specifically aimed at comparing groups, analyzing survival before the initiation of different treatment strategies could provide further insights into baseline comparability.

Strengths of this study include its large study population with a substantial number of patients choosing CKM, long follow-up time, and the real-world setting with careful documentation of the treatment decision moment. This allowed for survival analysis starting from the point of decision. Another strength is the stratification of patients choosing CKM as either suitable or less suitable for dialysis. This distinction helps clinicians better assess whether the reported survival outcomes may be applicable to a patient, assuming a similar clinical setting. However, a key limitation of this study is the missing comorbidity data, which complicates the ability to assess the relevance of our finding to patients in practice. In addition, we were unable to determine what proportion of patients categorized as less suitable for dialysis had an absolute contraindication to dialysis.

Older patients represent a growing proportion of patients with end-stage kidney disease, with 55% of patients starting KRT in Europe aged 65 years or older.^31^ Previous research has highlighted that older patients with advanced CKD often prioritize maintaining independence in activities of daily living over survival and that factors beyond survival, such as quality of life, treatment burden, the impact on loved ones, and costs, play a key role in the decision to choose CKM.^3^^,^^32^ These findings underscore the importance of understanding each patient’s individual values and priorities. It is crucial to discuss a broad range of patient-centered outcomes during counseling for KRT to ensure that treatment decisions align with what matters most to the patient. Future research should also focus on these broader outcomes to better capture the diverse factors influencing patients’ decisions and well-being.

In this cohort of older patients with kidney failure, patients choosing CKM who were suitable for dialysis experienced longer survival outcomes than has been previously reported. These results emphasize the importance of evaluating dialysis suitability when assessing survival outcomes in CKM. This supports more individualized shared decision making in older adults with advanced kidney disease.
