# Supplementary material for: Survival of Older Adults Choosing Dialysis or Conservative Kidney Management, Stratified by Suitability for Dialysis
Source: Kidney Med. 2026 Jun 12;8(8):101430. doi: 10.1016/j.xkme.2026.101430 (PMC13416626; doi:10.1016/j.xkme.2026.101430)

Supplementary Material

Table S1: Frequency of considerations identified in health records underlying the nephrologists' assessment of patients being less suitable for dialysis

Considerations	Frequency
Suspected or diagnosed malignancy	8
Malignancy + frailty or other comorbidities	3
Cognitive impairment	8
Cognitive impairment + other comorbidities	6
CVA + frailty or other comorbidities	6
Advanced age	4
Advanced age + frailty or other comorbidities	24
Cardiac disease	8
Cardiac disease + frailty	21
Cardiac disease + advanced age	5
Cardiac disease + advanced age + frailty	16
Pulmonary disease	2
Pulmonary disease + cardiac disease	5
Frailty	11
Frailty + other comorbidities	6
Other comorbidities	3
Unknown	3

Figure S1: Flow chart of electronic health record screening and inclusion

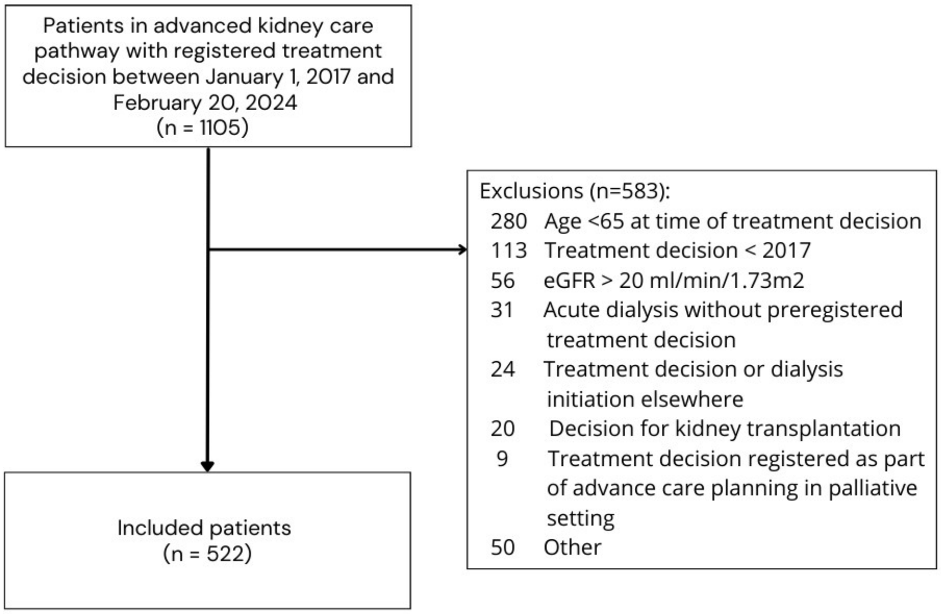

Figure S2: Kaplan Meier estimates of survival for patients choosing dialysis, patients suitable for dialysis who chose CKM and patients less suitable for dialysis, measured from A) first eGFR measurement ≤ 15 ml/min/1.73m^2, and B) first eGFR measurement ≤ 10 ml/min/1.73m^2.

A

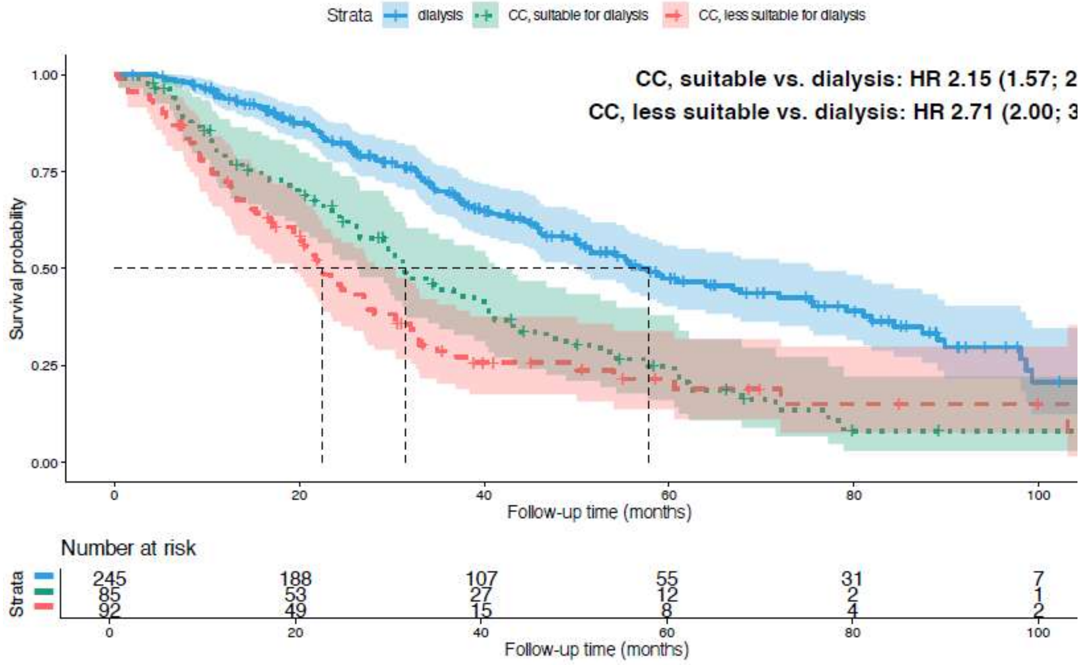

B

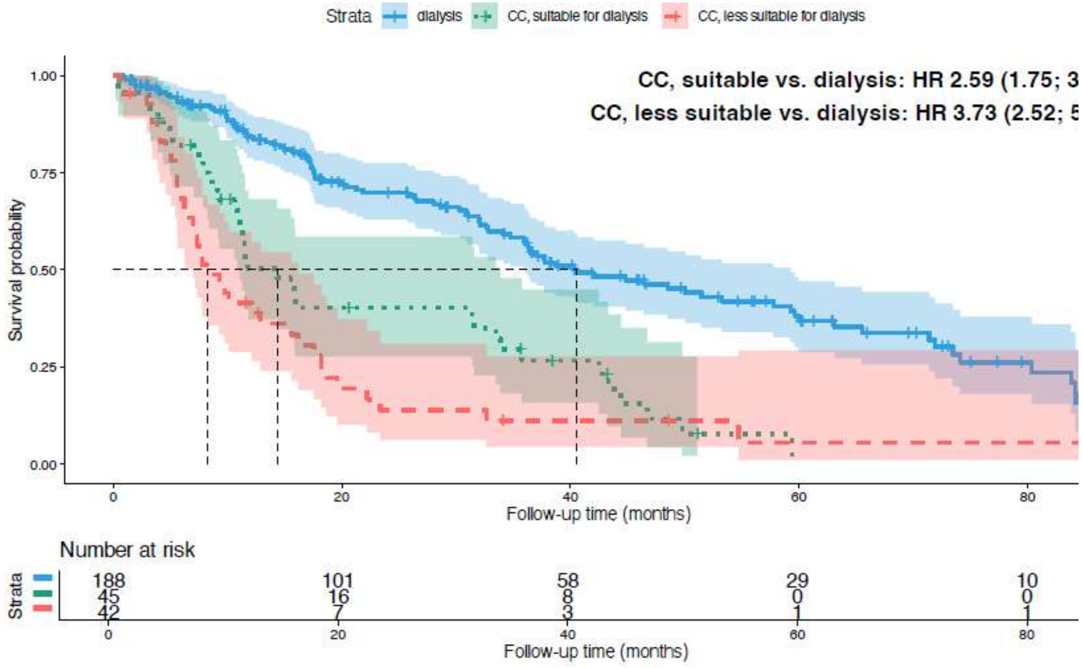

Figure S3: Kaplan Meier curve of survival from moment of treatment decision in patients choosing HD and PD

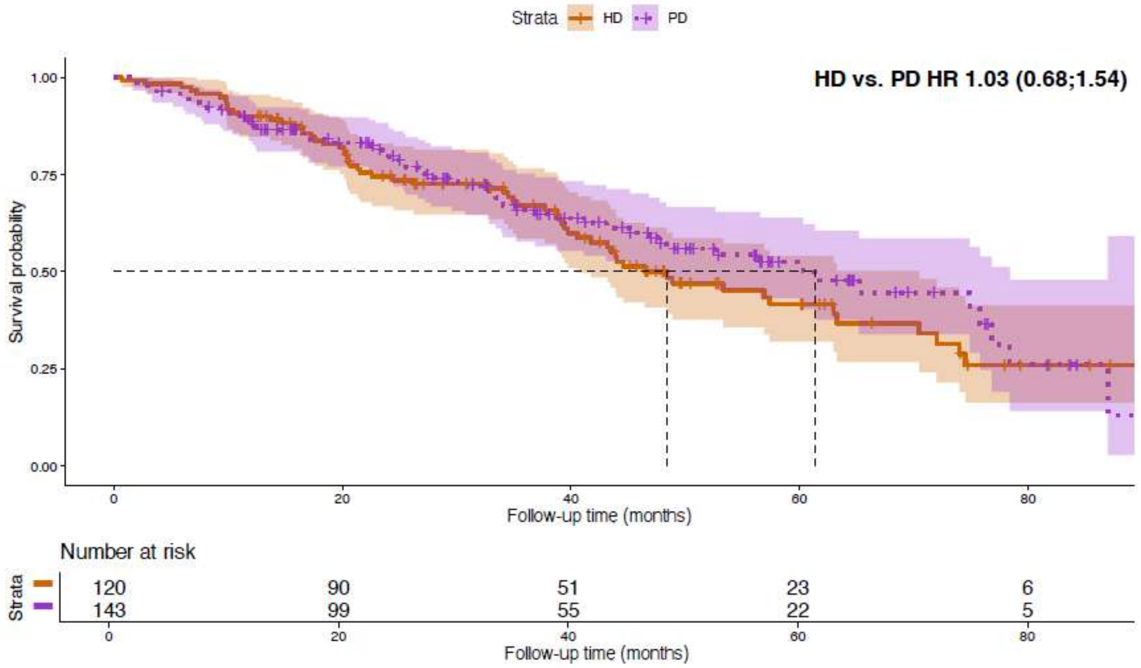

Supplement: Supplementary File (PDF) — Table S1; Figures S1-S3 [file mmc1.pdf]
